# Sb_2_O_3_/Ag/Sb_2_O_3_ Multilayer Transparent Conducting Films For Ultraviolet Organic Light-emitting Diode

**DOI:** 10.1038/srep41250

**Published:** 2017-01-25

**Authors:** Chunyan Song, Nan Zhang, Jie Lin, Xiaoyang Guo, Xingyuan Liu

**Affiliations:** 1 State Key Laboratory of Luminescence and Applications, Changchun Institute of Optics, Fine Mechanics and Physics, Chinese Academy of Sciences, Changchun 130033, China

## Abstract

A novel UV transparent conducting films based on Sb_2_O_3_/Ag/Sb_2_O_3_ (SAS) structure, which were prepared by an electron-beam thermal evaporation at room temperature. This SAS exhibits excellent electrical, optical and stable properties. Especially for UV region, the SAS has high transmittance of 80% at 306 nm and 92% at 335 nm, meanwhile achieving low sheet resistance ( ≤ 10 Ω sq^−1^). The UV OLED based on the SAS show competitive device performance. The UV OLED obtains the peak of UV electroluminescence at 376 nm and shows a very high maximum EQE of 4.1% with the maximum output power density of 5.18 mW cm^−2^. These results indicate that the potential of SAS applications in deep UV transparent electrodes and large-scale flexible transparent electronics.

Transparent conducting films (TCFs) have been widely used in optoelectronics fields including flat panel displays (FPD)^1^, organic light-emitting diodes (OLEDs)^2^, thin film solar cells^3^, and photodetectors^4^. Currently commercial TCFs are transparent conducting oxides (TCOs) including indium tin oxides (ITOs) and fluorine-doped tin oxides (FTOs) films. Recently, several emerging materials such as conducting polymers^5^, carbon nanotubes^6^, graphene^7^, and metallic nanowires have shown favorable electrical conductivity and transparency as those of ITO^8^, which plays important role in devices. However, all the above conducting materials are only transparent in the visible region.

With the development of ultraviolet (UV) optoelectronic devices such as UV photodetectors^9^, UV electrically pumped laser and UV LEDs^10^, there are an enhanced need for the UV TCFs. While, UV transparency means a wide energy gap (E_g_) for the TCOs. Most intrinsic wide band gap materials are close to insulation, showing poor conductivity. It is theoretically feasible to obtain UV TCFs by the introduction of shallow donor level in wide band gap materials through doping. However, up to now, few UV TCFs were reported. By doping β-Ga_2_O_3_ (E_g_ = 4.9 eV) with Sn, Masahiro Orita *et al*.^11^,^12^ obtained a transmittance of 50% at deep UV 248 nm with a conductivity of 1 S cm^−1^ and electron concentration of 1.43 × 10^19^ cm^−3^, these parameters are significantly lower compared to the TCFs in the visible region.

According to previous studies^13^, a dielectric-metal-dielectric (DMD) structure can be optimized through tuning the thickness of dielectric and metal layers to achieve high transparency in the visible region as well as high conductivity^3^,^14^. Nowadays, the DMD electrodes have been applied to organic light-emitting diodes^15^ and organic PV cells^16^, and the properties of these devices are comparable to those devices based on ITO electrodes. Generally, DMD TCFs have advantageous of excellent conductivity properties than most single layer TCFs and an easy tunable transparent area by adjusting the thickness of the dielectric film. By employing a wider bandgap material, it is possible to achieve UV TCFs with high transparency and conductivity. Antimony trioxide (Sb_2_O_3_) is a UV transparent semiconductor material with a wide E_g_ of 3.6 eV^17^, which has higher electrical conductivity than that of other UV transparent semiconductors^18^,^19^. Therefore, we introduce Sb_2_O_3_ into DMD structure in this work, and develop a deep UV Sb_2_O_3_/Ag/Sb_2_O_3_ (SAS) TCFs under room temperature, and successfully make it with high adjustable transmittance from UV to visible region, with low resistivity at an order of 10^−5^. A UV OLEDs has been obtained based on SAS anode, which shows superior performance with EL Peak at 376 nm in the UV region.

## Results

**Figure 1:**
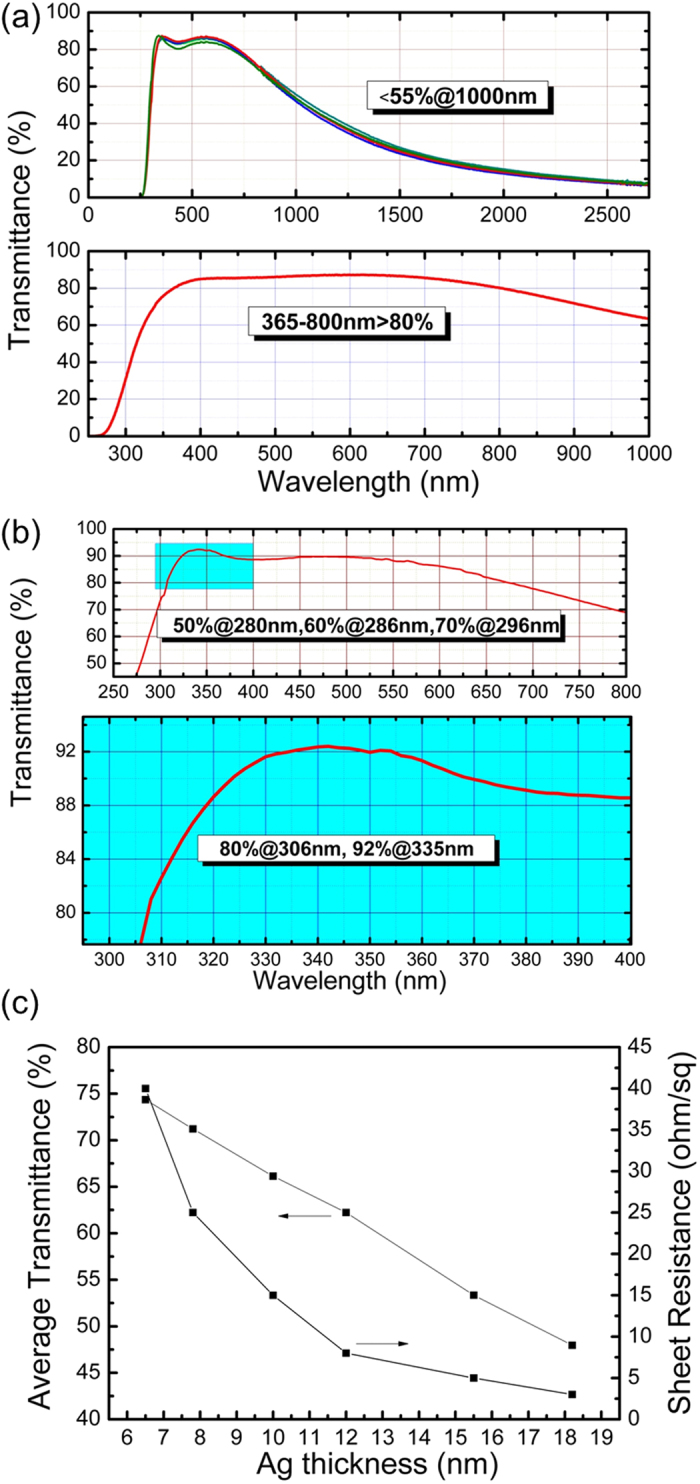
The optical and electrical properties of SAS. (**a**) The transmittance spectra of a series of SAS. (**b**) The deep UV transmittance spectra of SAS. (**c**) Average transmittance (300–380 nm) and sheet resistance of the SAS electrodes as a function of the Ag thickness. The structure of the electrodes is 35 nm/x (Ag thickness) nm/40 nm.

Figure 1a shows 250–2500 nm transmission spectrum of a series of SAS, they have high transmission from the UV to visible region, and low transmission in infrared region (IR), respectively. However, those regions are not fixed, they vary with the SAS each layer thicknesses. Through optical designing^13^,^15^, the SAS films can realize high transmission in different regions (Figure S1). We developed SAS with the structure of 51 nm/18 nm/32 nm, and achieved high transmittance in deep UV region (Fig. 1b). In TCO, deep UV transparent is quite dependent on wide E_g_, which contradicts with good electron properties. In this SAS films, due to the middle high conductive Ag layer is parallel to the Sb_2_O_3_ layers, the Hall carrier concentration is 7.722 × 10^21^ cm^−3^, the mobility is 21.92 cm^2^V^−1^s^−1^, the resistivity is 3.688 × 10^−5 ^Ω cm, the sheet resistance is 8 Ω sq^−1^, and the work function is −5.22 eV. The plasma resonance frequency is proportional to the carrier concentrations. The SAS carrier concentration is about one order bigger than TCO carrier concentration, thus it has a lower transmission (higher reflection) in IR region (Fig. 1a). It prevents absorbing thermal irradiation, which is good for many optoelectronic devices. Figure 1c shows the change in measured sheet resistance and average transmittance (λ = 300–380 nm) of SAS electrodes as a function of Ag thickness. The SAS sheet resistance decreases dramatically, from 40 Ω/sq to 3.5 Ω/sq, with increasing Ag thickness. This result indicates that the Ag thickness determines the electrical properties of the SAS electrodes. SAS electrodes with Ag layer thickness of less than 8 nm show higher sheet resistance because of the discontinuous Ag island growth^20^,^21^. With the thickness of Ag layer increased to 18 nm, the average transmittance (λ = 300–380 nm) of SAS decreases gradually, which can result from the high reflectance of Ag layer.

**Figure 2:**
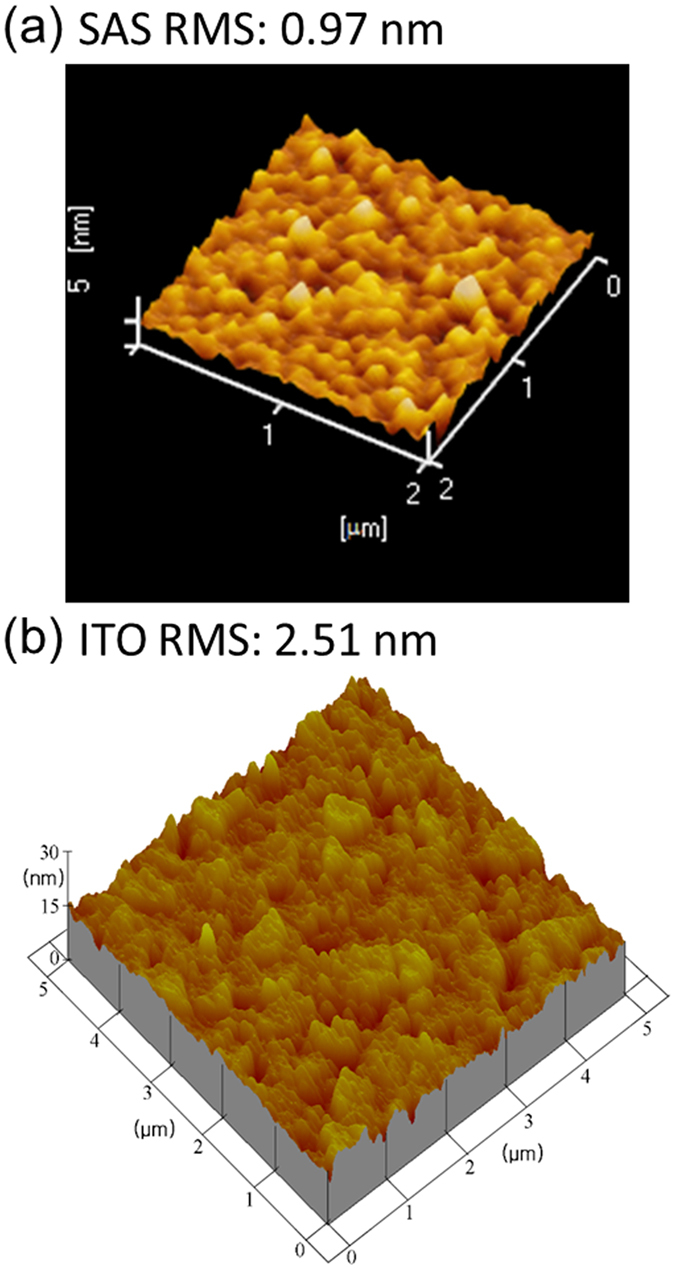
The characterization of surface morphology of SAS. AFM micrographs of SAS (ITO) sample.

Surface morphology and roughness are also very important for transparent electrodes, when they are applied in optoelectronics devices. As shown in Fig. 2, the root-mean-square roughness of the SAS structure deposited on glass is only 0.97 nm, which is much lower than that of sputter-derived commercial ITO (2.51 nm) and might contribute to the carrier injection and transportation performance^21^. The high quality surface morphologies can result from the process of electron-beam evaporator deposition, which affects the film deposition, oxidation, and crystallization simultaneously^22^.

**Figure 3:**
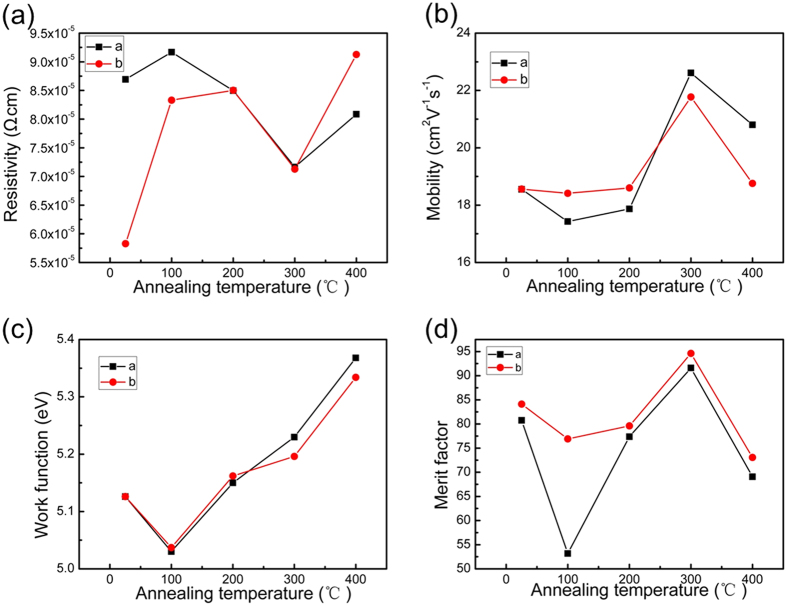
The properties of SAS under different annealing temperatures. (**a**) Resistivity, (**b**) mobility, (**c**) work function and (**d**) merit factor of the SAS films before and after annealing at different temperatures for 30 min in air. The structure of SAS sample a: 55 nm/14 nm/45 nm; sample b: 53 nm/14.5 nm/46 nm.

TCFs that can sustain high temperature are rare. In TCFs of DMD structure with Ag as the central metal layer, thermal stability is a research difficulty. They tend to have problems with thermal and time stability, because Ag is easy to have chemical reaction with ambient air, generates Ag_2_S. We studied the electrical property of SAS under different annealing temperatures. We prepared two SAS samples, with the structures a: 55 nm/14 nm/45 nm; b: 53 nm/14.5 nm/46 nm, and studied their resistivity, mobility, work function and film figure of merit against annealing temperatures under 100, 200, 300, and 400 °C (Fig. 3).

From the Fig. 3a and b we can see that, both the resistivity and the carrier mobility of two samples shown the optimal performance under 300 °C annealing temperature, they got worse when the temperature increased to 400 °C. Also, we noticed that under 100 °C, almost all the electrical properties are the worst. Ag film structure has a change between 100 ~ 200 °C according to literature^23^. In the heat treatment conditions, the size of the particles etc. factors that affect the electrical performances are changing and 100 °C might just be the worst situation. From the Fig. 3c, we can see that the work function of two samples increase with annealing temperature after 100 °C. From Bardeen’s theory^24^, we know that the interaction strength of a double layer of atomic dimension at the semiconductor surface acts a comparable role to that space charge, which can determine the work function. Due to oxygen vacancy conductivity, Sb_2_O_3_ is a metal oxide semiconductor. Under thermal treatment, Sb_2_O_3_ reacts with the ambient air, the charge dipole layer of the surface has been changed, which may cause the altering of work functions. Work function is a critical factor for many microelectronics, a proper work function can reduce the contact barrier significantly, thus improve the device performance effectively. However, compare to the abundant semiconductor materials, the current available different work function TCFs are few. The changeable work function of SAS will match many more materials’ energy level, which will be helpful in many devices.

Generally we use figure of merit Ф_*TC*_ to evaluate whether a TCF is good or not, the bigger, the better. According to Haacke function^25^:


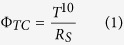


where T denotes the TCF film transmittance, R_s_ represents the film sheet resistance. Similarly, two SAS samples achieved the best performance under 300 °C annealing temperature (Fig. 3d).

Both SAS sample still have good performance under high temperature 400 °C, SAS is the first DMD TCF that can endure such a high temperature. Sb_2_O_3_ has very good thermal stability, the dense Sb_2_O_3_ film can protect Ag layer against oxidant with ambient air, and therefore SAS can sustain high temperature. We suggest that the DMD TCFs’ thermal stability is mainly dependent on the dielectric layer, hence adopting proper materials, we can obtain high thermal stability TCFs.

**Figure 4:**
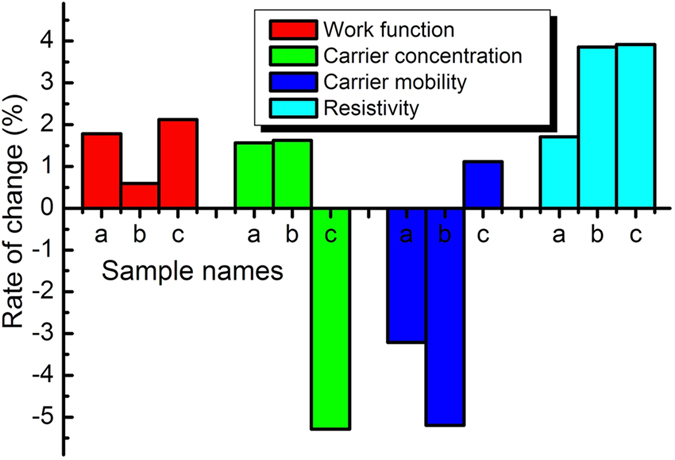
Time stability of SAS. Time stability of sample a, b and c. Sample a: 12 months and 10days, Sample b: 12 months and one week and Sample c: 10 months.

Time stability is another key factor for the applications of DMD TCFs. We compared three different SAS samples (a, b and c) of their electrical properties after 12 months and 10 days, 12 months and one week, and 10 months, respectively. They all had very good stability, the rate of change are all below 6% (Fig. 4). All the samples were stored in the atmosphere.

**Figure 5:**
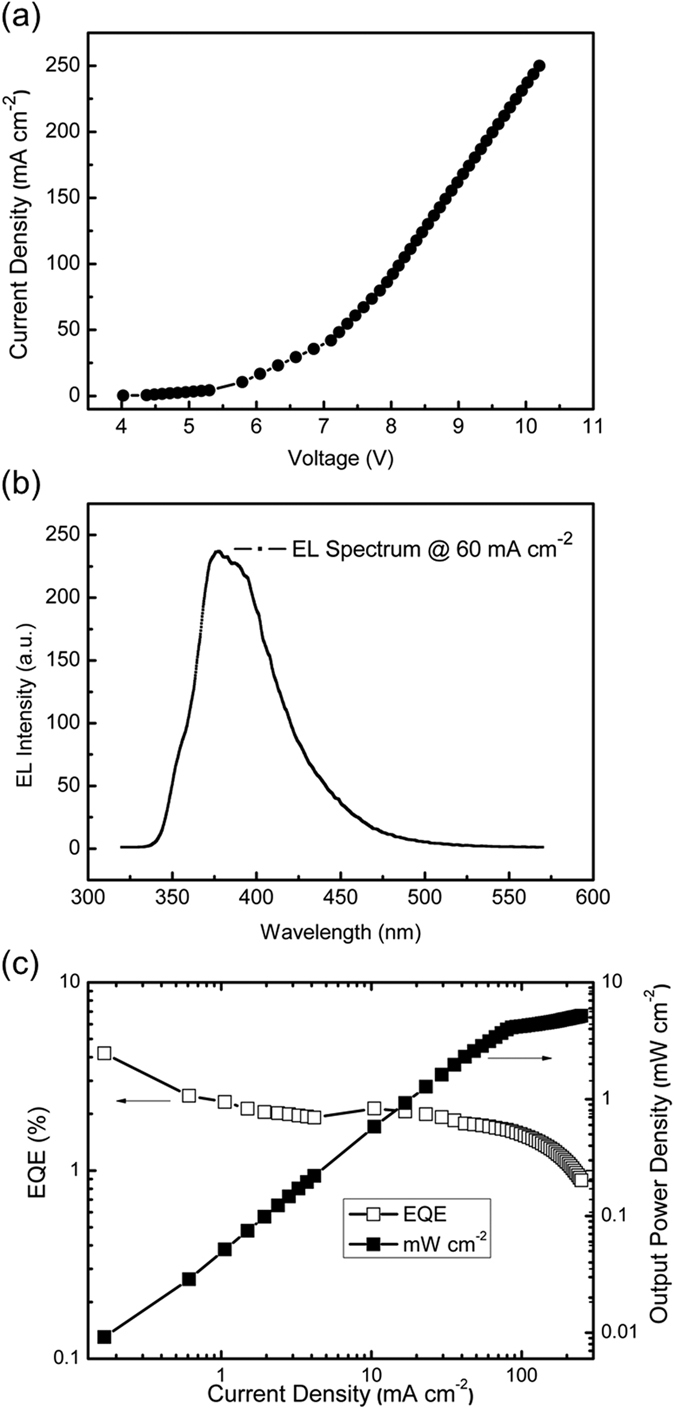
Characteristics of the UV OLED based on SAS. (**a**) Current density–voltage characteristics of OLED. (**b**) EL spectrum of OLEDs at 60 mA cm^−2^. (**c**) EQE and output power density changing with the injection current density.

The SAS as UV transparent anode was used in OLED. The effective area is etched as 1 mm × 1 mm. The device structure is Glass/SAS/WO_3_/TcTa/CBP/PBD/TPBI/LiF/Al. The J-V characters were recorded by Keithley 2611 SourceMeter, as shown in Fig. 5a. The device exhibits attractive UV emission with the Peak at 376 nm (Fig. 5b). Owing to the high work function that matches the energy level better, and the lower sheet resistance, the turn on and operation voltage are relative lower compare to the reported results^26^. The output power and EL spectra were measured by PM310E (thorlabs), Avantes fiber spectrometer. The maximum external quantum efficiency (EQE) of 4.1% has been demonstrated with the maximum output power density of 5.18 mW cm^−2^ (in Fig. 5c), which is one of the best results among the research of UV OLED to our knowledge.

In conclusion, we have developed SAS TCFs under room temperature. SAS have extraordinary performance that can have high transparency from deep UV to visible region, meanwhile obtaining low sheet resistance. By varying layer thicknesses, the high transparent regions are changeable, and the SAS work functions are adjustable under annealing. It is the first UV TCF that has such properties that SAS can sustain 400 °C temperature and longtime stability. The SAS was used in UV OLED, which achieved UV electroluminescence at 376 nm and exhibited a very high maximum EQE of 4.1% with the maximum output power density of 5.18 mW cm^−2^. The outstanding optical and electrical properties of the SAS films enable numerous applications including deep UV transparent electrodes and large-scale flexible transparent electronics.

## Methods

### Sb_2_O_3_/Ag/Sb_2_O_3_ Multilayer Films Fabrication and Characterization

The thin layers of Sb_2_O_3_/Ag/ Sb_2_O_3_ multilayer films were deposited on glass and polymer substrate using an electron-beam evaporator under room temperature. For both Sb_2_O_3_ layers, the oxygen gas was introduced upstream at 15 ~ 16.9 SCCM to insure that the vacuum pressure was among 1.9 ~ 2.2 × 10^−2^ Pa. The evaporation rate of Sb_2_O_3_ material was about 0.06 ~ 0.1 nm s^−1^, and the optimal one is 0.08 nm s^−1^. The Ag material was evaporated below 1.0 × 10^−3^ Pa with a rate of 1 nm s^−1^. Optical transmittance spectra of SAS were evaluated with a Shimadzu UV-3101PC spectrophotometer. Carrier concentrations, resistivities and Hall carrier mobility were carried out by Hall Effect measurements with the maximum magnetic filed applied of 0.55 T. Film thicknesses were measured with an Ambios XP-1 surface profiler. The surface resistance was measured using the four-point probe method with a surface resistivity meter. The work functions of samples were investigated by a KP Technology Ambient Kelvin probe system package.

### UV OLED Fabrication and Characterization

A glass substrate coated with thin layers of Sb_2_O_3_/Ag/Sb_2_O_3_ multilayer films, which technological parameters have optimized. The SAS film was cleaned with acetone, methanol, distilled water, and isopropyl alcohol sequentially. The device structure is Glass/SAS/WO_3_ (1 nm)/TcTa (20 nm)/CBP (30 nm)/PBD (20 nm)/TPBI (35 nm)/LiF (1 nm)/Al (100 nm). All organic layers were sequentially deposited on to the substrate without breaking the vacuum at a pressure of 5 × 10^−4^ Pa. using thermal evaporation equipment. The deposition rates were controlled from 2.0 to 3.0 Å s^−1^ for organic materials and 0.1 Å s^−1^ for lithium fluorine (LiF). Finally, the aluminum cathode was deposited at a rate of 10 Å s^−1^. The deposition rates were controlled with a quartz crystal monitor and corrected by footstep machine simultaneously. The devices had emission areas of 1 mm × 1 mm. Electroluminescence spectra were taken using an Avantes USB 3648 spectrometer. Power-current-voltage curves were measured using a system incorporating a Thorlabs PM320E powermeter and a Keithley 2611 source-measure unit. The method of direct measurement of EQE was introduced by S.R. Forrest and D.D.C^27^. Bradley, using a placed probe (Thorlabs) in contact with the active pixel, making sure the device under test underfilled the probe area. The emission from the device was then measured as output power while the current through the device was monitored simultaneously. These two quantities were then used to calculate the EQE (as photons per electron) by converting the power signal to emitted photons and the device current to electrons. All measurements were performed in air at room temperature.

## Additional Information

**How to cite this article**: Song, C. *et al*. Sb_2_O_3_/Ag/Sb_2_O_3_ Multilayer Transparent Conducting Films For Ultraviolet Organic Light-emitting Diode. *Sci. Rep.* **7**, 41250; doi: 10.1038/srep41250 (2017).

**Publisher's note:** Springer Nature remains neutral with regard to jurisdictional claims in published maps and institutional affiliations.

## Supplementary Material

Supplementary Information
