# Supplementary material for: Sb2O3/Ag/Sb2O3 Multilayer Transparent Conducting Films For Ultraviolet Organic Light-emitting Diode
Source: Sci Rep. 2017 Jan 25;7:41250. doi: 10.1038/srep41250 (PMC5264597; doi:10.1038/srep41250)
Supplement: Supplementary Information [file srep41250-s1.doc]

Supporting Information for Scientific Reports

**Sb_2_O_3_/Ag/Sb_2_O_3_ Multilayer Transparent Conducting Films For Ultraviolet Organic Light-emitting Diode**

Chunyan Song^1^, Nan Zhang^1,*^, Jie Lin^1^, Xiaoyang Guo^1^, and Xingyuan Liu^1,*^

^1^State Key Laboratory of Luminescence and Applications, Changchun Institute of Optics, Fine Mechanics and Physics, Chinese Academy of Sciences, Changchun 130033, China

Corresponding Author

* E-mail: [zhangn@ciomp.ac.cn](mailto:zhangn@ciomp.ac.cn); [liuxy@ciomp.ac.cn](mailto:liuxy@ciomp.ac.cn)


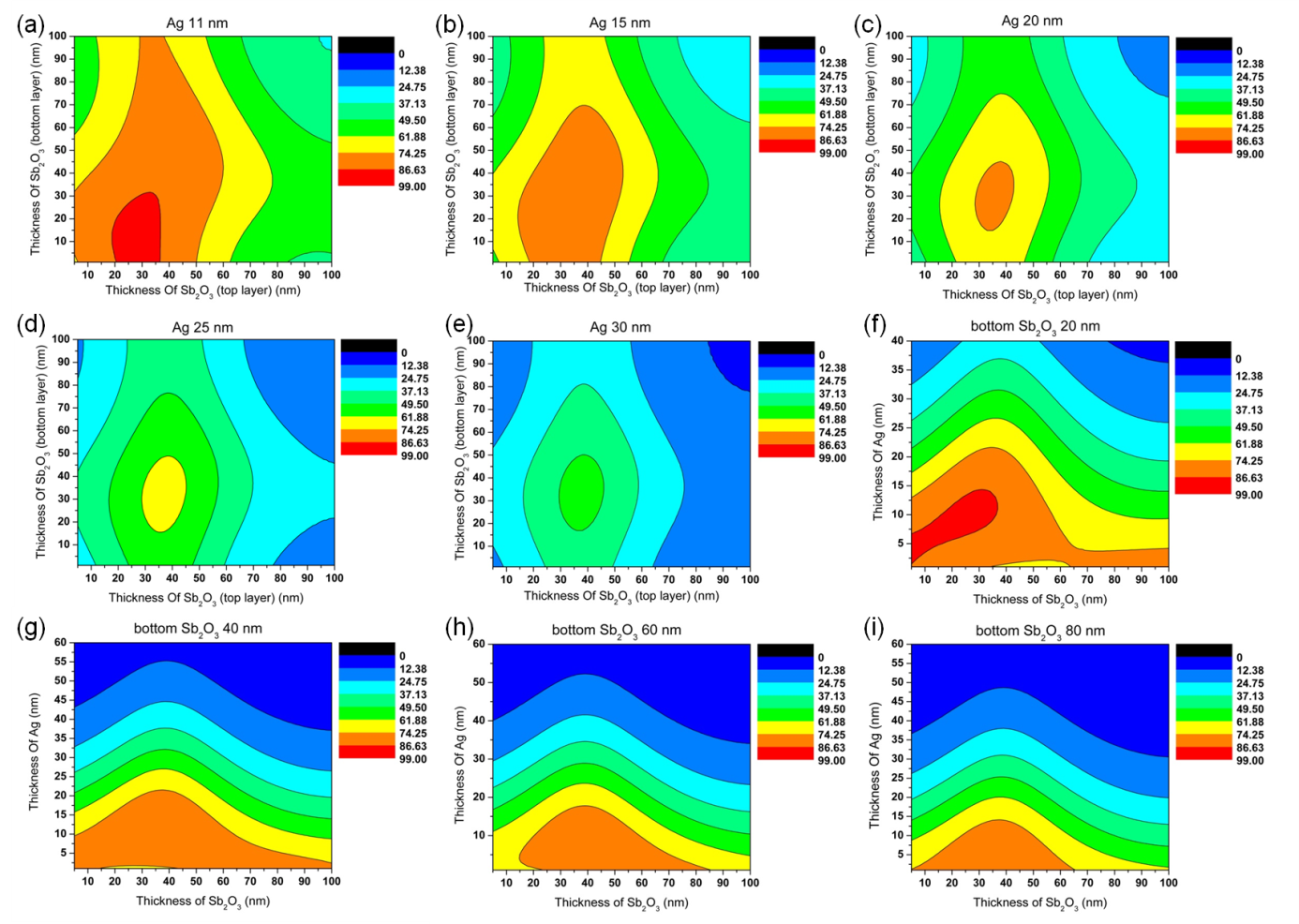


**Figure S1.** Calculated transmittance of the SAS structure at a wavelength of 550 nm as a function of Ag and Sb_2_O_3_ thicknesses.
